# Correction: Hydrogel microfibers with perfusable folded channels for tissue constructs with folded morphology

**DOI:** 10.1039/c9ra90025j

**Published:** 2019-04-04

**Authors:** Yupeng Liu, Peidi Xu, Zhe Liang, Ruoxiao Xie, Mingyu Ding, Hongxia Liu, Qionglin Liang

**Affiliations:** MOE Key Laboratory Bioorganic Phosphorous Chemistry & Chemical Biology, Beijing Key Laboratory of Microanalytical Methods & Instrumentation, Department of Chemistry, Tsinghua University Beijing 100084 China liangql@tsinghua.edu.cn; The State Key Laboratory of Chemical Oncogenomics, The Graduate School at Shenzhen, Tsinghua University Shenzhen 518055 China

## Abstract

Correction for ‘Hydrogel microfibers with perfusable folded channels for tissue constructs with folded morphology’ by Yupeng Liu *et al.*, *RSC Adv.*, 2018, **8**, 23475–23480.

The authors regret that incorrect details were given for ref. 49 in the original article. The correct version of ref. 49 is given below.

The Royal Society of Chemistry apologises for these errors and any consequent inconvenience to authors and readers.

## Supplementary Material
